# Eprosartan mesylate, an angiotensin II receptor antagonist

**DOI:** 10.1107/S1600536811006659

**Published:** 2011-03-02

**Authors:** Jing-Jing Qian, Xiu-Rong Hu, Jianming Gu, Su-Xiang Wu

**Affiliations:** a College of Pharmaceutical Science, Zhejiang Chinese Medical University, Hangzhou, Zhejiang 310053, People’s Republic of China; b Center of Analysis and Measurement, Zhejiang University, Hangzhou, Zhejiang 310028, People’s Republic of China

## Abstract

The title compound, eprosartan mesylate {systematic name: 2-butyl-1-(4-carb­oxy­benz­yl)-5-[(*E*)-2-carb­oxy-3-(thio­phen-2-yl)prop-1-en­yl]-1*H*-imidazol-3-ium methane­sulfonate}, C_23_H_25_N_2_O_4_S^+^·CH_3_O_3_S^−^, one of the angiotensin II-receptor antagonists, is effective in regulating hypertension, induced or exacerbated by angiotensin II, and in the treatment of congestive heart failure, renal failure and glaucoma. In the eprosartan residue, which appears in this crystal in the cationic imidazolium form, the benzene ring plane is almost orthogonal to that of the imidazole ring, making a dihedral angle of 87.89 (2)°. The thio­phene ring forms dihedral angles of 66.54 (2) and 67.12 (2)° with the benzene and imidazole rings, respectively. The imidazolium NH group and the H atom of the aromatic carboxyl group participate in hydrogen bonds with the the O atoms of the anion, thus forming centrosymmetric aggregates made up of two cations and two anions each. The second carboxyl group further links the above-mentioned aggregates through a conventional centrosymmetric hydrogen-bonding motif into infinite chains along [011].

## Related literature

For applications of eprosartan mesylate in medicine, see: Punzi & Punzi (2005 ▶); Punzi *et al.* (2004 ▶); Hillaert *et al.* (2003 ▶). For the crystal structures of other eprosartan derivatives, see: Wu *et al.* (2009 ▶); Sheng *et al.* (1999 ▶). For the preparation of eprosartan mesylate, see Bandi *et al.* (2010 ▶). 
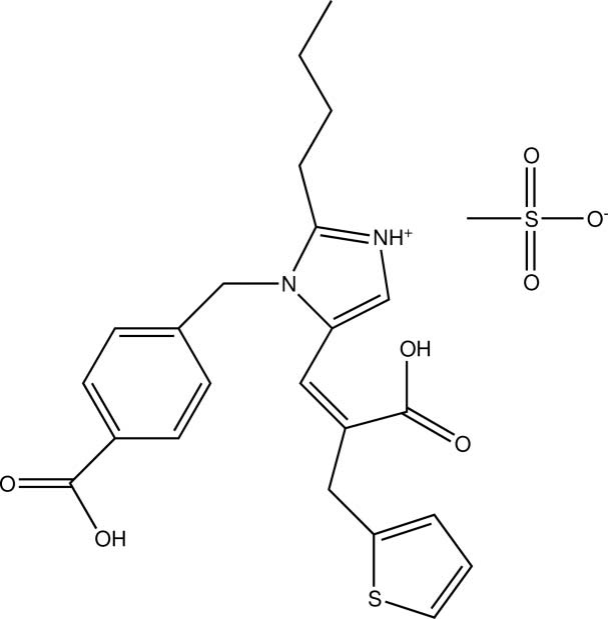


## Experimental

### 

#### Crystal data

C_23_H_25_N_2_O_4_S^+^·CH_3_O_3_S^−^
*M* *_r_* = 520.60
Triclinic, 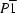

*a* = 8.6635 (4) Å
*b* = 12.6935 (7) Å
*c* = 13.6679 (8) Å
α = 112.700 (2)°
β = 101.386 (1)°
γ = 96.718 (1)°
*V* = 1327.97 (12) Å^3^
*Z* = 2
Mo *K*α radiation
μ = 0.25 mm^−1^
*T* = 193 K
0.48 × 0.34 × 0.16 mm

#### Data collection

Rigaku R-AXIS-RAPID/ZJUG diffractometer
Absorption correction: multi-scan (*ABSCOR*; Higashi, 1995 ▶) *T*_min_ = 0.879, *T*_max_ = 0.961
10590 measured reflections
4689 independent reflections
3248 reflections with *I* > 2σ(*I*)
*R*_int_ = 0.022

#### Refinement

*R*[*F* ^2^ > 2σ(*F* ^2^)] = 0.083
*wR*(*F* ^2^) = 0.225
*S* = 0.99
4689 reflections
318 parameters
12 restraints
H-atom parameters constrained
Δρ_max_ = 1.35 e Å^−3^
Δρ_min_ = −0.62 e Å^−3^

### 

Data collection: *PROCESS-AUTO* (Rigaku, 2006 ▶); cell refinement: *PROCESS-AUTO*; data reduction: *CrystalStructure* (Rigaku, 2007 ▶); program(s) used to solve structure: *SHELXS97* (Sheldrick, 2008 ▶); program(s) used to refine structure: *SHELXL97* (Sheldrick, 2008 ▶); molecular graphics: *ORTEP-3 for Windows* (Farrugia, 1997 ▶); software used to prepare material for publication: *WinGX* (Farrugia, 1999 ▶).

## Supplementary Material

Crystal structure: contains datablocks I, global. DOI: 10.1107/S1600536811006659/ya2135sup1.cif

Structure factors: contains datablocks I. DOI: 10.1107/S1600536811006659/ya2135Isup2.hkl

Additional supplementary materials:  crystallographic information; 3D view; checkCIF report

## Figures and Tables

**Table 1:** Hydrogen-bond geometry (Å, °)

*D*—H⋯*A*	*D*—H	H⋯*A*	*D*⋯*A*	*D*—H⋯*A*
N2—H2*A*⋯O5	0.88	1.86	2.697 (5)	158
O1—H1⋯O2^i^	0.84	1.80	2.628 (5)	171
O3—H3⋯O6^ii^	0.84	1.78	2.597 (5)	162

Symmetry codes: (i) 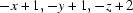; (ii) 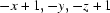.

**Fig. 1.**
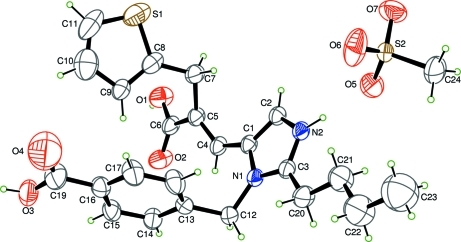
Molecular structure of the title compound; displacement ellipsoids are drawn at the 40% probability level. H atoms are shown as small circles of arbitrary radius.

**Fig. 2.**
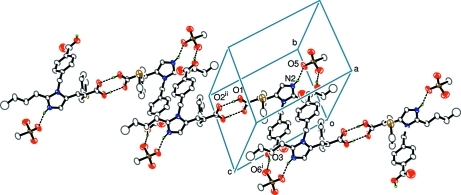
Crystal packing of the title compound viewed approximately down the a axis. H-bonds are shown as dashed lines.

## supplementary crystallographic information

### Comment

Angiotensin-II-receptor antagonists are safe and effective agents for the treatment of hypertension and heart failure, either alone, or in conjunction with hydrochlorothiazide, a thiazide diuretic (Hillaert *et al.*, 2003; Punzi & Punzi, 2005). The title compound, eprosartan mesylate, is one of the highly selective, orally active, non-peptide angiotensin-II-receptor antagonists, which has low likelihood for undesirable drug interactions. It is reported that eprosartan mesylate may be potentially attractive in the treatment of elderly patients who are often on multiple drug regimens (Punzi *et al.*,2004).

The crystal structure of eprosartan in the form of monohydrate of neutral molecule has been recently published (Wu *et al.*, 2009). Although the title compound was known already for more than a decade (Sheng *et al.*,1999), its crystal structure has not yet been reported and represents the subject of the present paper.

The asymmetric unit, comprising eprosartan cation and mesylate anion, is shown in Fig.1. Geometric parameters of the cation are comparable to that of the neutral eprosartan (Wu *et al.*, 2009). Phenyl ring plane is almost orthogonal to imidazole plane, corresponding dihedral angle being equal to 87.89 (2)°. Thiophene plane forms dihedral angles of 66.54 (2)° and 67.12 (2)°, with phenyl and imidazole planes, respectively. Conformation of the molecule in the structure of eprosartan hydrate (Wu *et al.*, 2009) shows substantial differences; in particular the dihedral angle between thiophene and imidazole planes in hydrate structure is much smaller [24.78 (2)°].

The imidazolium NH-group and carboxyl H atom bound to O3 participate in H-bonds with the the oxygen atoms of the anion thus forming centrosymmetric aggregates made up of two cations and two anions each (Fig.2). The second carboxyl H atom (bound to O1) is involved in centrosymmetric H-bonding motive, typical for carboxyl structures; in this way the above mentioned aggregates get linked into infinite chains stretching along the [011] direction.

### Experimental

Methyl-4-[(2-*n*-butyl-5-formyl-1*H*-imidazol-1-yl) methyl] benzoate (10 g), was added to a mixture of 135 ml of n-heptane and 15 ml of dichloromethane at room temperature. The reaction mixture was maintained in Dean Stark apparatus at 343–353 K for the duration of 15–30 min. Then piperidinium acetate catalyst (2.8 g of piperidine and 5.55 g of acetic acid) dissolved in the mixture of 8.5 ml n-heptane and 1.5 ml dichloromethane was added to the reaction mixture followed by addition of 2-thiophene-2-yl-methylmalonic acid monoethyl ester (17.3 g). Reaction temperature was maintained at 343–353 K for 20 h. After reaction completion, cooling to room temperature, ethanol and de-ionized water were added, and pH was adjusted to 1 using 1*M* HCl. The layers were separated, and the aqueous layer was washed with n-heptane. Then pH of the aqueous layer was adjusted to 6 with 1*M* NaOH and the solution was extracted with toluene. Combined organic layers were concentrated under vacuum, the residue was dissolved in 130 ml of ethanol, and solution of NaOH (13.5 g of NaOH in 65 ml of water) was added and stirred for 1–2 h. Thereafter, pH of the reaction mixture was adjusted to 4.5–5 with 1*M* HCl. The precipitated solid was filtered, washed with water and dried under vacuum to yield 11 g of eprosartan (Bandi *et al.*, 2010). 10 g of eprosartan was dissolved in 150 ml of isopropyl alcohol at room temperature. 6.8 g of methane sulfonic acid was added to the clear solution which was then stirred for about 2 h. The solid was filtered, washed with isopropyl alcohol and dried to yield 10 g of eprosartan mesylate, which was recrystallized from ethanol solution, giving colorless crystals of the title compound suitable for X-ray diffraction.

### Refinement

The difference density map indicated the presence of a possible H atom at the N2 atom, thus confirming proton transfer from mesylate to imidazole. Subsequently, this H atom was placed in calculated position with N—H 0.88 Å and refined as riding with *U*_iso_(H) = 1.2*U*_eq_(N). All other H atoms were placed in calculated positions as well with O—H 0.84 Å, and C—H bonds of 0.99 Å for methylene, 0.98 Å for methyl, and 0.95Å for aromatic H atoms; all H-atoms were included in the refinement in riding model approximation, with *U*_iso_(H) = 1.2*U*_eq_ of the carrying atom (1.5 *U*_eq_ in case of OH and methyl groups). Temperature factors of the O4 and C23 atoms were restrained to represent isotropic behavior [ISOR 0.003 according to *SHELXL97* (Sheldrick, 2008)]. The highest peak in the residual difference map [1.35 e Å^-3^] is at a distance of 0.97 Å from the O4 atom.

### Crystal data

**Table:**

C_23_H_25_N_2_O_4_S^+^·CH_3_O_3_S^−^	*Z* = 2
*M**_r_* = 520.60	*F*(000) = 548
Triclinic, *P*1	*D*_x_ = 1.302 Mg m^−^^3^
Hall symbol: -P 1	Mo *K*α radiation, λ = 0.71073 Å
*a* = 8.6635 (4) Å	Cell parameters from 8503 reflections
*b* = 12.6935 (7) Å	θ = 3.0–27.4°
*c* = 13.6679 (8) Å	µ = 0.25 mm^−^^1^
α = 112.700 (2)°	*T* = 193 K
β = 101.386 (1)°	Platelet, colorless
γ = 96.718 (1)°	0.48 × 0.34 × 0.16 mm
*V* = 1327.97 (12) Å^3^	

### Data collection

**Table:**

Rigaku R-AXIS-RAPID/ZJUG diffractometer	4689 independent reflections
Radiation source: rotating anode	3248 reflections with *I* > 2σ(*I*)
graphite	*R*_int_ = 0.022
Detector resolution: 10.00 pixels mm^-1^	θ_max_ = 25.0°, θ_min_ = 3.0°
ω scans	*h* = −9→10
Absorption correction: multi-scan (*ABSCOR*; Higashi, 1995)	*k* = −15→14
*T*_min_ = 0.879, *T*_max_ = 0.961	*l* = −16→16
10590 measured reflections	

### Refinement

**Table:**

Refinement on *F*^2^	Primary atom site location: structure-invariant direct methods
Least-squares matrix: full	Secondary atom site location: difference Fourier map
*R*[*F*^2^ > 2σ(*F*^2^)] = 0.083	Hydrogen site location: inferred from neighbouring sites
*wR*(*F*^2^) = 0.225	H-atom parameters constrained
*S* = 0.99	*w* = 1/[σ^2^(*F*_o_^2^) + (0.0839*P*)^2^ + 3.6024*P*] where *P* = (*F*_o_^2^ + 2*F*_c_^2^)/3
4689 reflections	(Δ/σ)_max_ = 0.001
318 parameters	Δρ_max_ = 1.35 e Å^−^^3^
12 restraints	Δρ_min_ = −0.62 e Å^−^^3^

### Special details

**Table:**

Geometry. All e.s.d.'s (except the e.s.d. in the dihedral angle between two l.s. planes) are estimated using the full covariance matrix. The cell e.s.d.'s are taken into account individually in the estimation of e.s.d.'s in distances, angles and torsion angles; correlations between e.s.d.'s in cell parameters are only used when they are defined by crystal symmetry. An approximate (isotropic) treatment of cell e.s.d.'s is used for estimating e.s.d.'s involving l.s. planes.
Refinement. Refinement of *F*^2^ against ALL reflections. The weighted *R*-factor *wR* and goodness of fit *S* are based on *F*^2^, conventional *R*-factors *R* are based on *F*, with *F* set to zero for negative *F*^2^. The threshold expression of *F*^2^ > σ(*F*^2^) is used only for calculating *R*-factors(gt) *etc*. and is not relevant to the choice of reflections for refinement. *R*-factors based on *F*^2^ are statistically about twice as large as those based on *F*, and *R*- factors based on ALL data will be even larger.

### Fractional atomic coordinates and isotropic or equivalent isotropic displacement parameters (Å^2^)

**Table:**

	*x*	*y*	*z*	*U*_iso_*/*U*_eq_	
O2	0.4103 (5)	0.4459 (4)	0.8655 (3)	0.0774 (12)	
O7	1.0612 (4)	0.4213 (4)	0.3064 (4)	0.0925 (14)	
O5	0.7861 (5)	0.4244 (5)	0.3127 (4)	0.0928 (14)	
O6	0.8704 (7)	0.2450 (4)	0.2308 (5)	0.1191 (19)	
C24	0.8490 (8)	0.3725 (6)	0.1275 (5)	0.0821 (17)	
H24A	0.7348	0.3368	0.0906	0.123*	
H24B	0.9153	0.3287	0.0829	0.123*	
H24C	0.8707	0.4537	0.1366	0.123*	
O4	0.3022 (10)	−0.1534 (7)	0.6016 (7)	0.165 (3)	
S2	0.89587 (14)	0.36913 (11)	0.25638 (10)	0.0517 (4)	
S1	0.9345 (2)	0.2510 (2)	0.84615 (15)	0.0991 (7)	
N1	0.3712 (4)	0.3219 (3)	0.4681 (3)	0.0437 (8)	
N2	0.5741 (4)	0.3570 (3)	0.4086 (3)	0.0449 (8)	
H2A	0.6329	0.3611	0.3640	0.054*	
O1	0.6716 (4)	0.4864 (4)	0.9480 (3)	0.0751 (11)	
H1	0.6375	0.5105	1.0044	0.113*	
C1	0.5038 (5)	0.3802 (4)	0.5597 (3)	0.0431 (10)	
C3	0.4180 (5)	0.3063 (4)	0.3763 (3)	0.0443 (10)	
C2	0.6297 (5)	0.4018 (4)	0.5203 (3)	0.0450 (10)	
H2	0.7370	0.4409	0.5625	0.054*	
O3	0.1466 (5)	−0.0801 (4)	0.7013 (4)	0.0770 (11)	
H3	0.1472	−0.1412	0.7116	0.116*	
C4	0.4876 (5)	0.4100 (4)	0.6710 (3)	0.0473 (10)	
H4	0.3906	0.4325	0.6850	0.057*	
C12	0.2101 (5)	0.2783 (4)	0.4736 (4)	0.0511 (11)	
H12A	0.1341	0.2465	0.3995	0.061*	
H12B	0.1716	0.3436	0.5233	0.061*	
C5	0.5937 (6)	0.4090 (4)	0.7544 (4)	0.0499 (11)	
C15	0.1446 (6)	0.0995 (4)	0.6343 (4)	0.0561 (12)	
H15	0.0945	0.1024	0.6910	0.067*	
C13	0.2129 (5)	0.1834 (4)	0.5152 (4)	0.0463 (10)	
C6	0.5533 (6)	0.4495 (5)	0.8623 (4)	0.0552 (12)	
C14	0.1433 (6)	0.1868 (4)	0.5977 (4)	0.0553 (12)	
H14	0.0936	0.2498	0.6301	0.066*	
C7	0.7515 (6)	0.3701 (5)	0.7518 (4)	0.0636 (14)	
H7A	0.7659	0.3455	0.6766	0.076*	
H7B	0.8415	0.4365	0.8025	0.076*	
C17	0.2873 (7)	0.0043 (5)	0.5060 (5)	0.0708 (15)	
H17	0.3372	−0.0585	0.4737	0.085*	
C9	0.6218 (6)	0.1805 (4)	0.7697 (4)	0.0523 (11)	
H9	0.5112	0.1735	0.7374	0.063*	
C8	0.7559 (7)	0.2699 (5)	0.7850 (4)	0.0625 (14)	
C16	0.2179 (6)	0.0083 (4)	0.5895 (4)	0.0547 (11)	
C18	0.2848 (7)	0.0905 (4)	0.4692 (5)	0.0649 (14)	
H18	0.3328	0.0867	0.4115	0.078*	
C20	0.3160 (6)	0.2441 (5)	0.2616 (4)	0.0629 (13)	
H20A	0.2265	0.2843	0.2530	0.075*	
H20B	0.2678	0.1640	0.2497	0.075*	
C19	0.2231 (8)	−0.0823 (5)	0.6307 (6)	0.0813 (18)	
C10	0.6885 (12)	0.1052 (6)	0.8128 (6)	0.100 (2)	
H10	0.6227	0.0400	0.8130	0.120*	
C22	0.2867 (9)	0.1741 (8)	0.0603 (5)	0.114 (3)	
H22A	0.1978	0.2157	0.0554	0.137*	
H22B	0.2389	0.0937	0.0480	0.137*	
C21	0.3989 (7)	0.2353 (6)	0.1744 (4)	0.0809 (17)	
H21A	0.4492	0.3149	0.1863	0.097*	
H21B	0.4860	0.1923	0.1804	0.097*	
C11	0.8466 (12)	0.1306 (7)	0.8526 (6)	0.103 (2)	
H11	0.9040	0.0853	0.8817	0.123*	
C23	0.3652 (14)	0.1675 (11)	−0.0273 (10)	0.167 (4)	
H23A	0.2858	0.1265	−0.0990	0.251*	
H23B	0.4520	0.1248	−0.0243	0.251*	
H23C	0.4102	0.2466	−0.0171	0.251*	

### Atomic displacement parameters (Å^2^)

**Table:**

	*U*^11^	*U*^22^	*U*^33^	*U*^12^	*U*^13^	*U*^23^
O2	0.070 (2)	0.131 (3)	0.049 (2)	0.048 (2)	0.0280 (18)	0.042 (2)
O7	0.048 (2)	0.137 (4)	0.085 (3)	0.002 (2)	0.006 (2)	0.049 (3)
O5	0.091 (3)	0.141 (4)	0.088 (3)	0.056 (3)	0.056 (2)	0.066 (3)
O6	0.170 (5)	0.078 (3)	0.125 (4)	0.013 (3)	0.024 (4)	0.071 (3)
C24	0.096 (4)	0.108 (5)	0.060 (3)	0.030 (4)	0.025 (3)	0.049 (3)
O4	0.186 (4)	0.158 (3)	0.180 (4)	0.050 (3)	0.067 (3)	0.089 (3)
S2	0.0486 (7)	0.0683 (8)	0.0548 (7)	0.0151 (6)	0.0172 (5)	0.0407 (6)
S1	0.0965 (13)	0.1373 (17)	0.0840 (12)	0.0633 (12)	0.0275 (10)	0.0554 (12)
N1	0.046 (2)	0.054 (2)	0.043 (2)	0.0174 (17)	0.0175 (16)	0.0288 (17)
N2	0.049 (2)	0.057 (2)	0.0415 (19)	0.0199 (17)	0.0198 (16)	0.0274 (17)
O1	0.073 (2)	0.110 (3)	0.0413 (19)	0.031 (2)	0.0161 (18)	0.028 (2)
C1	0.050 (2)	0.048 (2)	0.039 (2)	0.018 (2)	0.0169 (19)	0.0216 (19)
C3	0.050 (2)	0.051 (2)	0.041 (2)	0.019 (2)	0.0165 (19)	0.025 (2)
C2	0.051 (2)	0.050 (2)	0.038 (2)	0.016 (2)	0.0135 (19)	0.0209 (19)
O3	0.090 (3)	0.078 (3)	0.084 (3)	0.020 (2)	0.028 (2)	0.053 (2)
C4	0.055 (3)	0.054 (3)	0.042 (2)	0.022 (2)	0.019 (2)	0.025 (2)
C12	0.046 (2)	0.064 (3)	0.056 (3)	0.016 (2)	0.018 (2)	0.036 (2)
C5	0.056 (3)	0.060 (3)	0.046 (3)	0.022 (2)	0.021 (2)	0.029 (2)
C15	0.062 (3)	0.063 (3)	0.056 (3)	0.016 (2)	0.028 (2)	0.032 (2)
C13	0.041 (2)	0.054 (3)	0.047 (2)	0.009 (2)	0.0128 (19)	0.024 (2)
C6	0.065 (3)	0.072 (3)	0.044 (3)	0.030 (3)	0.022 (2)	0.033 (2)
C14	0.063 (3)	0.058 (3)	0.061 (3)	0.026 (2)	0.031 (2)	0.031 (2)
C7	0.061 (3)	0.095 (4)	0.058 (3)	0.032 (3)	0.027 (2)	0.046 (3)
C17	0.084 (4)	0.059 (3)	0.091 (4)	0.031 (3)	0.052 (3)	0.035 (3)
C9	0.064 (3)	0.049 (3)	0.052 (3)	0.022 (2)	0.019 (2)	0.026 (2)
C8	0.077 (3)	0.088 (4)	0.040 (3)	0.047 (3)	0.028 (2)	0.032 (3)
C16	0.054 (3)	0.048 (3)	0.066 (3)	0.011 (2)	0.019 (2)	0.027 (2)
C18	0.078 (4)	0.063 (3)	0.072 (3)	0.024 (3)	0.045 (3)	0.033 (3)
C20	0.060 (3)	0.085 (4)	0.041 (3)	0.016 (3)	0.011 (2)	0.026 (3)
C19	0.095 (4)	0.062 (3)	0.118 (5)	0.044 (3)	0.055 (4)	0.049 (4)
C10	0.137 (7)	0.075 (4)	0.093 (5)	0.026 (5)	0.044 (5)	0.033 (4)
C22	0.103 (5)	0.170 (8)	0.045 (3)	0.005 (5)	0.016 (3)	0.029 (4)
C21	0.082 (4)	0.104 (5)	0.045 (3)	0.008 (3)	0.014 (3)	0.024 (3)
C11	0.151 (7)	0.104 (5)	0.089 (5)	0.075 (6)	0.044 (5)	0.060 (4)
C23	0.168 (5)	0.173 (5)	0.158 (5)	0.032 (3)	0.045 (3)	0.067 (3)

### Geometric parameters (Å, °)

**Table:**

O2—C6	1.245 (6)	C15—C16	1.378 (7)
O7—S2	1.414 (4)	C15—C14	1.381 (6)
O5—S2	1.421 (4)	C15—H15	0.9500
O6—S2	1.455 (4)	C13—C14	1.370 (6)
C24—S2	1.747 (5)	C13—C18	1.388 (7)
C24—H24A	0.9800	C14—H14	0.9500
C24—H24B	0.9800	C7—C8	1.509 (7)
C24—H24C	0.9800	C7—H7A	0.9900
O4—C19	1.191 (8)	C7—H7B	0.9900
S1—C11	1.668 (8)	C17—C18	1.369 (7)
S1—C8	1.698 (5)	C17—C16	1.378 (7)
N1—C3	1.343 (5)	C17—H17	0.9500
N1—C1	1.398 (5)	C9—C10	1.423 (9)
N1—C12	1.468 (5)	C9—C8	1.448 (7)
N2—C3	1.334 (5)	C9—H9	0.9500
N2—C2	1.365 (5)	C16—C19	1.465 (7)
N2—H2A	0.8800	C18—H18	0.9500
O1—C6	1.277 (6)	C20—C21	1.484 (7)
O1—H1	0.8400	C20—H20A	0.9900
C1—C2	1.353 (6)	C20—H20B	0.9900
C1—C4	1.459 (6)	C10—C11	1.320 (10)
C3—C20	1.482 (6)	C10—H10	0.9500
C2—H2	0.9500	C22—C21	1.506 (8)
O3—C19	1.268 (7)	C22—C23	1.470 (12)
O3—H3	0.8400	C22—H22A	0.9900
C4—C5	1.320 (6)	C22—H22B	0.9900
C4—H4	0.9500	C21—H21A	0.9900
C12—C13	1.518 (6)	C21—H21B	0.9900
C12—H12A	0.9900	C11—H11	0.9500
C12—H12B	0.9900	C23—H23A	0.9800
C5—C6	1.491 (6)	C23—H23B	0.9800
C5—C7	1.509 (6)	C23—H23C	0.9800
			
S2—C24—H24A	109.5	C8—C7—C5	111.0 (4)
S2—C24—H24B	109.5	C8—C7—H7A	109.4
H24A—C24—H24B	109.5	C5—C7—H7A	109.4
S2—C24—H24C	109.5	C8—C7—H7B	109.4
H24A—C24—H24C	109.5	C5—C7—H7B	109.4
H24B—C24—H24C	109.5	H7A—C7—H7B	108.0
O7—S2—O5	116.2 (3)	C18—C17—C16	120.4 (5)
O7—S2—O6	109.4 (3)	C18—C17—H17	119.8
O5—S2—O6	112.5 (3)	C16—C17—H17	119.8
O7—S2—C24	107.5 (3)	C10—C9—C8	106.3 (5)
O5—S2—C24	106.6 (3)	C10—C9—H9	126.9
O6—S2—C24	103.8 (3)	C8—C9—H9	126.9
C11—S1—C8	92.4 (4)	C9—C8—C7	127.8 (4)
C3—N1—C1	109.3 (3)	C9—C8—S1	112.1 (4)
C3—N1—C12	126.4 (4)	C7—C8—S1	120.0 (4)
C1—N1—C12	124.1 (3)	C15—C16—C17	119.0 (4)
C3—N2—C2	110.5 (3)	C15—C16—C19	120.4 (5)
C3—N2—H2A	124.7	C17—C16—C19	120.7 (5)
C2—N2—H2A	124.7	C17—C18—C13	120.9 (5)
C6—O1—H1	109.5	C17—C18—H18	119.6
C2—C1—N1	106.1 (4)	C13—C18—H18	119.6
C2—C1—C4	132.7 (4)	C3—C20—C21	115.9 (4)
N1—C1—C4	121.2 (4)	C3—C20—H20A	108.3
N2—C3—N1	106.7 (4)	C21—C20—H20A	108.3
N2—C3—C20	126.8 (4)	C3—C20—H20B	108.3
N1—C3—C20	126.6 (4)	C21—C20—H20B	108.3
C1—C2—N2	107.3 (4)	H20A—C20—H20B	107.4
C1—C2—H2	126.3	O4—C19—O3	121.1 (7)
N2—C2—H2	126.3	O4—C19—C16	120.6 (7)
C19—O3—H3	109.5	O3—C19—C16	118.3 (5)
C5—C4—C1	126.8 (4)	C11—C10—C9	116.6 (7)
C5—C4—H4	116.6	C11—C10—H10	121.7
C1—C4—H4	116.6	C9—C10—H10	121.7
N1—C12—C13	110.8 (3)	C21—C22—C23	113.8 (7)
N1—C12—H12A	109.5	C21—C22—H22A	108.8
C13—C12—H12A	109.5	C23—C22—H22A	108.8
N1—C12—H12B	109.5	C21—C22—H22B	108.8
C13—C12—H12B	109.5	C23—C22—H22B	108.8
H12A—C12—H12B	108.1	H22A—C22—H22B	107.7
C4—C5—C6	116.9 (4)	C22—C21—C20	112.9 (5)
C4—C5—C7	126.8 (4)	C22—C21—H21A	109.0
C6—C5—C7	116.4 (4)	C20—C21—H21A	109.0
C16—C15—C14	120.5 (4)	C22—C21—H21B	109.0
C16—C15—H15	119.7	C20—C21—H21B	109.0
C14—C15—H15	119.7	H21A—C21—H21B	107.8
C14—C13—C18	118.6 (4)	C10—C11—S1	112.7 (6)
C14—C13—C12	120.9 (4)	C10—C11—H11	123.7
C18—C13—C12	120.5 (4)	S1—C11—H11	123.7
O2—C6—O1	123.5 (4)	C22—C23—H23A	109.5
O2—C6—C5	120.0 (4)	C22—C23—H23B	109.5
O1—C6—C5	116.5 (4)	H23A—C23—H23B	109.5
C13—C14—C15	120.6 (4)	C22—C23—H23C	109.5
C13—C14—H14	119.7	H23A—C23—H23C	109.5
C15—C14—H14	119.7	H23B—C23—H23C	109.5
			
C3—N1—C1—C2	1.7 (5)	C16—C15—C14—C13	0.9 (8)
C12—N1—C1—C2	177.7 (4)	C4—C5—C7—C8	−120.2 (5)
C3—N1—C1—C4	179.3 (4)	C6—C5—C7—C8	59.3 (6)
C12—N1—C1—C4	−4.7 (6)	C10—C9—C8—C7	178.9 (5)
C2—N2—C3—N1	2.1 (5)	C10—C9—C8—S1	0.0 (5)
C2—N2—C3—C20	−177.6 (4)	C5—C7—C8—C9	30.1 (7)
C1—N1—C3—N2	−2.3 (5)	C5—C7—C8—S1	−151.1 (4)
C12—N1—C3—N2	−178.2 (4)	C11—S1—C8—C9	0.7 (4)
C1—N1—C3—C20	177.3 (4)	C11—S1—C8—C7	−178.3 (4)
C12—N1—C3—C20	1.5 (7)	C14—C15—C16—C17	−1.2 (8)
N1—C1—C2—N2	−0.4 (5)	C14—C15—C16—C19	178.2 (5)
C4—C1—C2—N2	−177.6 (4)	C18—C17—C16—C15	0.7 (9)
C3—N2—C2—C1	−1.1 (5)	C18—C17—C16—C19	−178.7 (6)
C2—C1—C4—C5	−39.5 (8)	C16—C17—C18—C13	0.2 (9)
N1—C1—C4—C5	143.6 (5)	C14—C13—C18—C17	−0.5 (8)
C3—N1—C12—C13	111.2 (5)	C12—C13—C18—C17	−179.7 (5)
C1—N1—C12—C13	−64.1 (5)	N2—C3—C20—C21	1.8 (8)
C1—C4—C5—C6	176.7 (4)	N1—C3—C20—C21	−177.8 (5)
C1—C4—C5—C7	−3.8 (8)	C15—C16—C19—O4	−171.1 (7)
N1—C12—C13—C14	131.3 (5)	C17—C16—C19—O4	8.3 (11)
N1—C12—C13—C18	−49.5 (6)	C15—C16—C19—O3	6.2 (9)
C4—C5—C6—O2	23.4 (7)	C17—C16—C19—O3	−174.4 (6)
C7—C5—C6—O2	−156.2 (5)	C8—C9—C10—C11	−1.0 (8)
C4—C5—C6—O1	−156.8 (5)	C23—C22—C21—C20	178.2 (8)
C7—C5—C6—O1	23.6 (7)	C3—C20—C21—C22	−178.4 (6)
C18—C13—C14—C15	0.0 (7)	C9—C10—C11—S1	1.6 (9)
C12—C13—C14—C15	179.2 (4)	C8—S1—C11—C10	−1.3 (6)

### Hydrogen-bond geometry (Å, °)

**Table:**

*D*—H···*A*	*D*—H	H···*A*	*D*···*A*	*D*—H···*A*
N2—H2A···O5	0.88	1.86	2.697 (5)	158
O1—H1···O2^i^	0.84	1.80	2.628 (5)	171
O3—H3···O6^ii^	0.84	1.78	2.597 (5)	162

Symmetry codes: (i) −*x*+1, −*y*+1, −*z*+2; (ii) −*x*+1, −*y*, −*z*+1.
